# Migration pattern and biochemical response characteristics of polylactic acid nanoparticles in pakchoi (*Brassica chinensis* L. cv. SuZhou) seedlings

**DOI:** 10.3389/fpls.2026.1718625

**Published:** 2026-01-30

**Authors:** Xinye Zhao, Qing Luo, Wenju Dai, Yongyao Deng, Ning Yang, Xu Zhu, Yixuan Zheng, Ying Li, Liangshan Feng

**Affiliations:** 1 Key Laboratory of Ecological Restoration of Regional Contaminated Environment, Ministry of Education, College of Environment, Shenyang University, Shenyang, China; 2 Plant Protection College, Shenyang Agricultural University, Shenyang, China; 3 Liaoning Academy of Agricultural Sciences, Shenyang, China

**Keywords:** antioxidant defense system, biodegradable plastics, migrate, osmotic control system, polylactic acid nanoplastics

## Abstract

Nanoplastics represent an emerging class of pollutants that infiltrate aquatic and terrestrial environments through diverse pathways, posing significant threats to ecosystems. However, research on the accumulation and translocation mechanisms of biodegradable nanoplastics in plants remains limited. In this investigation, pakchoi (*Brassica chinensis* L. cv. SuZhou) was exposed to fluorescently labeled polylactic acid nanoplastics (PLA-NPs) in hydroponic solutions with varying concentrations (20 mg/L, 50 mg/L) and particle sizes (170 nm, 330 nm) to investigate the migration, distribution patterns, and associated toxicological responses of PLA-NPs in pakchoi. Both microscopic imaging and fluorescence semi-quantitative analysis confirmed that PLA-NPs of both tested particle sizes can enter the root system via the apical meristem and primary root-lateral root junction. Furthermore, PLA-NPs with a smaller particle size (170 nm) and higher concentration (50 mg/L) are more readily absorbed and accumulated by roots, and subsequently translocated to aboveground tissues. When roots were exposed to PLA-NPs, the activities of superoxide dismutase, peroxidase, and catalase in pakchoi significantly decreased, while hydrogen peroxide and malondialdehyde levels increased. Concurrently, soluble sugar, soluble protein, and chlorophyll content also changed. Moreover, the magnitude of these changes increased with the increase in PLA-NPs particle size and concentration. Collectively, PLA-NPs accumulate in pakchoi seedling roots, translocate to aboveground tissues, and potentially posing certain risks to human health through the food chain.

## 1 Introduction

Plastic products are widely utilized due to their lightweight properties, durability, and cost-effectiveness (Jiang et al., 2019). However, they have also precipitated global environmental issues that pose substantial threats to ecosystems and human health (Aanesen et al., 2024; Dokl et al., 2024; Guo et al., 2024; Li and Liu, 2024; Zhang and Liu, 2018). Due to the extensive use of single-use plastics, enormous amounts of plastic waste have accumulated in various habitats. Accumulation in terrestrial ecosystems has reached alarming levels (Adamczyk et al., 2024; Sun et al., 2020), while marine ecosystems have similarly been impacted. It is estimated that approximately 311 million tons of plastic are produced globally each year, with a significant proportion allocated to packaging applications. Nevertheless, only about 14% of this plastic is recycled (Uday, 2023), as most is disposed of in landfills or released into the natural environment, exacerbating widespread plastic pollution. Projections suggest that global plastic consumption will rise to 1.231 billion tons by 2060 (OECD, 2022).

The environmental persistence of plastics represents a critical concern (Li et al., 2022). Once released, plastics undergo gradual fragmentation through biological, chemical, and physical mechanisms during use or in the surrounding environment, ultimately degrading into micro/nanoplastics (M/NPs,<5mm) (Ter Halle et al., 2016). These particles pose significant risks to living organisms. Substantial evidence confirms the phytotoxicity of M/NPs. For instance, increased concentrations of polystyrene microplastics (PS-MPs) significantly reduce plant germination rate and vigor. At the same time, levels of catalase (CAT), superoxide dismutase (SOD), hydrogen peroxide (H_2_O_2_), proline, soluble proteins, and soluble sugars are perturbed (Guo et al., 2022). Wang et al. (2022) demonstrated that M/NPs adhere to the surfaces of seeds and roots due to their small size and strong adsorption ability, which hinders germination, root growth, and nutrient and water uptake, ultimately restricting plant development.

Furthermore, Li et al. (2020) were the first to demonstrate that microplastics (MPs) can enter plants: by cultivating wheat and lettuce in nutrient solutions or soils containing polystyrene (PS) and polymethyl methacrylate (PMMA) microspheres, they observed accumulation of these microspheres in roots, stems, and leaves, and identified crevices at the edges of newly formed lateral roots as key sites for root uptake of MPs. Similarly, Liu et al. (2025a) employed red-fluorescent-labeled polystyrene nanoplastics (PS-NPs) and traced their transport within pakchoi, finding that PS-NPs entered the root xylem vascular bundles, translocated to petioles via transpiration, and ultimately reached leaves. In another study, onion seeds incubated in a 50 nm PS particle suspension for 72 hours contained plastic particles in various root tissues (e.g., stele, cortex, and epidermis), indicating that PS-NPs can penetrate multiple plant biological barriers to enter root cells (Giorgetti et al., 2020). Hua et al. (2024) found that PS-MPs can be absorbed by hydroponically grown lettuce through their root systems and transported via vascular bundles to edible parts such as stems and leaves. The size of microplastics directly influences their distribution, transport, and toxic effects within plants. Similarly, Zhou et al. (2021) observed that 20 nm PS particles localized to the intercellular spaces of rice roots.

In response to the challenges posed by conventional plastics, biodegradable plastics (BPs) have emerged as a promising alternative (Biopolymers, 2018; Sehrawat et al., 2025). Unlike traditional plastics(PP, PE, PS, and PVC), BPs consist of biobased or oil-based polymers that can be rapidly degraded by microbial enzymes (Weinstein et al., 2020). However, their actual degradation rate depends on environmental factors, including biotic and abiotic conditions (Fan et al., 2022). Studies have shown that industrial composting provides the most effective environment for their degradation (Tokiwa and Calabia, 2006). In natural settings, BPs released into the environment may similarly degrade into M/NPs due to the absence of ideal conditions, thereby impacting plants in a manner analogous to conventional plastics. For example, exposure to polybutylene adipate terephthalate (PBAT) biodegradable microplastics induces oxidative stress and activates plant defense mechanisms, disrupting the oxidative homeostasis that sustains growth and function (Adamczyk et al., 2024). The development of rice and buck-horn plantains was negatively impacted by the addition of 1% PBAT biodegradable MPs (Courtene-Jones et al., 2024; Irshad et al., 2024). Notably, some studies have reported no significant toxicity associated with plant growth for certain BPs (Kanwal et al., 2022; Palsikowski et al., 2018). Among these, polylactic acid (PLA) is produced by hydrolyzing starch from plants such as corn to yield glucose, which is then fermented to produce lactic acid and ultimately polymerized into PLA (Khare and Deshmukh, 2006). Statistics indicate that the global annual production capacity of PLA exceeded 600,000 tons in 2020 (Xin et al., 2020), garnering widespread attention. Lin et al. (2025) found that PLA-MPs (0.1–2.5% w/w) inhibited rice growth by reducing photosynthetic efficiency and suppressing root development. They also decreased soil nitrogen availability, altered phosphorus levels, reduced rhizosphere microbial α-diversity, and disrupted nutrient cycling. Despite their biodegradability, these particles remain detrimental to soil-crop systems. Another research team found that small-sized (25–38 μm) PLA-MPs significantly inhibited plant nitrogen uptake. This was due to their ammonium nitrogen immobilization rate far exceeding the mineralization rate, coupled with reduced abundance of nitrification functional genes, leading to decreased autotrophic nitrification rates and increased nitrate immobilization rates, posing a more pronounced threat to plants (Dan et al., 2025). An et al. (2025) revealed that PLA acts as an arsenic carrier, co-transporting arsenic through the xylem to accumulate in the leaf veins of rice seedlings, thereby increasing the total arsenic content. This dual contamination targets core metabolic hubs in the TCA cycle and phenylalanine synthesis, affecting antioxidant stress-related metabolites and impairing seedling growth. The primary focus of the current study has been on the toxicological effects of BPs on plants, whereas their migration patterns within plants remain underexplored. Thus, there is an urgent need for studies to elucidate the migration dynamics of BPs in plants.

Pakchoi (*Brassica chinensis* L. cv. SuZhou) was selected as the model plant for this study. As a typical leafy vegetable, it exhibits a shallow root system, short growth cycle, high vitamin and mineral content, low crude fiber, favorable taste, and high nutritional value, making it widely cultivated across East Asia, Northeast Asia, and Southeast Asia (Liu et al., 2025a, Liu et al., 2025b). Statistics indicate that pakchoi planting area in China accounts for 30–40% of the total vegetable planting area (Hanson et al., 2009). In this study, pakchoi was exposed to polylactic acid nanoplastics (PLA-NPs) of varying concentrations and particle sizes in hydroponic solution, aiming to elucidate the toxic effects of PLA-NPs on pakchoi and their migration patterns within the plant. This research addresses a critical gap in understanding the migration of BPs in plants, while providing a key foundation for in-depth insights into their environmental behavior and ecological risks.

## 2 Materials and methods

### 2.1 Materials and instruments

We bought Tween-20, Nile Red, and polylactic acid (Size = 3 mm, MW ∼80,000) from Shanghai Macklin Biochemical Technology Co., Ltd. (Shanghai, China). Dichloromethane, quartz sand, calcium carbonate powder, ethanol, and acetone were obtained from Tianjin Fuyu Fine Chemical Co., Ltd. (Tianjin, China). The Zetasizer Nano ZS90 laser particle sizer (LPSA) was provided by Malvern Panalytical (Malvern, UK). The Scientz-IID-ultrasonic cell disruptor (UCC) was purchased from Ningbo Xinzhi Biotechnology Co., Ltd. (Ningbo, China). The IRTracer-100 Fourier transform infrared spectrometer (FTIR) was provided by Shimadzu Corporation (Kyoto, Japan). The FV3000 laser scanning confocal microscope (LSCM) was supplied by Olympus Corporation (Tokyo, Japan). The F-4600 fluorescence spectrophotometer (FL) and S-4800 scanning electron microscopy (SEM) were provided by Hitachi, Ltd. (Tokyo, Japan). The L8-ultraviolet-visible (UV-Vis) spectrophotometer was provided by Shanghai Youke Instrumentation Co., Ltd. (Shanghai, China). All aqueous solutions were prepared using Milli-Q water (Deionized water 18 MΩ).

### 2.2 Preparation and characterization of PLA-NPs

The 170 nm and 330 nm fluorescently labeled PLA-NPs used in this experiment were developed based on the research group’s prior work on preparing unlabeled PLA particles (Zhang et al., 2024). Fluorescent labeling was introduced to enable visual tracking of particle migration within plants (Bretler and Margel, 2015). Preliminary experiments indicated that the fluorescent labeling process caused an increase in particle size. After comprehensive screening, unlabeled 100 nm and 200 nm particles demonstrated high labeling efficiency and good product stability. Their measured diameters after fluorescent labeling were 170 nm and 330 nm, respectively, thus establishing the target research sizes. The specific preparation procedure is as follows: Dissolve 0.1 g of PLA in 10 mL of dichloromethane, add 0.5 mL of Nile Red working solution (10 mg/mL, prepared in acetone), and sonicate to ensure complete dissolution, forming the organic phase. Slowly inject the organic phase into 100 mL aqueous phase containing Tween-20 surfactant. Ultrasonicate at 300 W for 30 min (1 second on, 1 second off), then stir at 1000 r/min for 12 h at room temperature to completely evaporate dichloromethane. The resulting suspension was centrifuged, washed (until no surfactant was detected in the supernatant), and freeze-dried to obtain the target fluorescently labeled PLA-NPs. The physicochemical properties of fluorescently labeled PLA-NPs were characterized using SEM, LPSA, FTIR, FL, and LSCM. The relative fluorescence intensity of fluorescently labeled PLA-NPs was analyzed and quantified using ImageJ software.

### 2.3 Exposure experiment

The pakchoi variety employed in this experiment was *Brassica chinensis* L. cv. SuZhou, which was acquired from the Liaoning Academy of Agricultural Sciences, has a germination rate of 98%. All seeds were stored in a sealed container in the dark at 4–8°C before the experiment. Seeds of similar plumpness were selected, surface-sterilized, and primed by soaking in 2% H_2_O_2_ solution for 10 minutes. After three thorough rinses with deionized water, it was immersed for an additional half hour. Seeds were sown at equal intervals in 9 cm Petri dishes (20 seeds per dish), with 5 mL of PLA-NPs suspensions of varying concentrations (20, 50 mg/L) (Sun et al., 2020) and particle sizes (170, 330 nm) added to each dish. After 4 days of germination, uniformly growing seedlings were selected and transplanted into beakers with 15 plants per beaker. The culture medium consisted of suspensions containing PLA-NPs of the specified concentrations and sizes, which were refreshed every 6 days to maintain consistent nanoplastics (NPs) levels over a total 28-day exposure period. Deionized water without PLA-NPs served as the control treatment, with three replicates per treatment.

### 2.4 Microscopic observation of pakchoi seedlings

Roots, stems, and petioles of pakchoi seedlings exposed for 7, 14, 21, and 28 days were sampled, rinsed with ultrapure water to remove surface impurities, and sonicated to eliminate potential residual PLA-NPs and potential root exudates adhering to the tissue surfaces; after sample pretreatment, plant tissue sections were prepared via manual sectioning (approximately 1 mm thick), and intact, wrinkle-free sections were selected for observation. These sections underwent XYZ three-dimensional scanning using an LSCM (excitation/emission wavelength: 536 nm/608 nm) to detect fluorescently labeled PLA-NPs in tissues. Semi-quantitative analysis was subsequently performed using ImageJ software. To ensure the reliability of experimental results, multiple quality control measures were implemented: (1) at least 3 plants were analyzed per treatment group, with 3 sections collected from distinct locations per plant; (2) consistent sectioning positions were selected to minimize location-induced variability; (3) Z-stack scanning technology was concurrently employed for correction (Robil et al., 2023). After section preparation, continuous multi-layer Z-stack scanning was performed with a single-slice thickness of 40 μm. ImageJ software was then used to stitch and calibrate the multi-layer scans, utilizing the superposition effect of multiple slices to compensate for potential human errors. This approach ensured compliance with the experimental requirement for result reliability.

### 2.5 Physiological indicators determination

After 28 days of exposure, samples were taken from each treatment group to assess the potential physiological and biochemical impacts of PLA-NPs on pakchoi seedlings. Measure fundamental indicators such as root length, germination rate, and fresh weight. The PLA-NPs induced response of the antioxidant defense system was analyzed by measuring the activities of SOD (BL5056-B), peroxidase (POD: BL5058-B), and CAT (BL4957-B), as well as the contents of H_2_O_2_ (BL4961-B) and malondialdehyde (MDA: BL7878-B). Soluble sugars (BL8082-B) and soluble proteins (BL5060-B) were used as indicators of osmoregulatory capacity. All the previously mentioned physiological and biochemical markers were evaluated using commercially available test kits (Shanghai Enzyme-linked Biotechnology Co., Ltd., Shanghai, China).

To determine chlorophyll content by spectrophotometry (Qin et al., 2025), take fresh plant leaves, add quartz sand, calcium carbonate powder, and 2–3 mL of 95% ethanol, then grind them into a homogeneous slurry. Add 10 mL of 95% ethanol and continue grinding until the tissue turns white; let stand for 3–5 minutes. Filter the extract through ethanol-moistened filter paper into a 25 mL brown volumetric flask. Wash the residue and filter paper until no green color remains, then dilute to volume and mix thoroughly. Transfer the extract into a 1 cm cuvette. Use 95% ethanol as the blank and measure the absorbance at wavelengths of 665 nm and 649 nm. Calculate chlorophyll content using Equations 1–3.

(1) $$\begin{array}{c} {\text{C}}_{\text{a}}=13.95A_{665}-6.88A_{649} \end{array}$$

(2) $$\begin{array}{c} {\text{C}}_{\text{b}}=24.96A_{649}-7.32A_{665} \end{array}$$

(3) $$\begin{array}{c} {\text{C}}_{\text{x}.\;c}=\frac{1000{\text{A}}_{470}-2.05C_{\text{a}}-114.8C_{\text{b}}}{245} \end{array}$$

In the formula, C_a_ represents chlorophyll a, C_b_ represents chlorophyll b, C_x.c_ represents carotenoids, and A_665_, A_649_, A_470_ represent the absorbance values at 665 nm, 649 nm, and 470 nm, respectively.

### 2.6 Statistical analysis

All data are presented as mean ± standard deviation and were analyzed using SPSS Statistics 27 software. Differences in measured indicators among treatment groups were assessed via one-way analysis of variance (ANOVA) followed by Duncan’s *post hoc* test. Repeated measures ANOVA was used to evaluate the quenching trend of fluorescently labeled PLA-NPs. Statistical significance was defined as *p<*0.05. Graphs were constructed using Origin 2024, and semi-quantitative analysis of microscopic images was conducted using ImageJ software.

## 3 Results and analysis

### 3.1 Characterization of PLA-NPs physicochemical properties

**Supplementary Figure S1** (a1) and S1(a2) show SEM images of 170 nm and 330 nm NPs, respectively, revealing that microspheres of both sizes are spherical with uniform particle size distribution and smooth surfaces. Zeta potential analysis results indicate that the surface charge of 170 nm NPs is -32.8333 ± 0.3786, while that of 330 nm NPs is -24.8 ± 1.0583, both carrying negative charges(**Supplementary Figure S1c**). **Supplementary Figure S1d** presents the FTIR spectra of the nanoparticles, with characteristic peaks including a CH_3_ stretching vibration at 3000 cm^-1^, a carbonyl (C=O) stretching vibration at 1759 cm^-1^, a CH_3_ bending vibration at 1457 cm^-1^, and a C-O stretching vibration at 1188 cm^-1^ (Wang et al., 2019); analysis of these peaks confirmed the substance as PLA. Fluorescence spectrophotometric analysis of the optical properties of the fluorescently labeled PLA-NPs identified maximum excitation/emission wavelengths of 536/608 nm, respectively (**Supplementary Figure S1e**). To further verify the fluorescence signal performance of PLA-NPs at these wavelengths, LSCM was employed. Results showed that PLA-NPs of both particle sizes emitted strong red fluorescence signals under the tested conditions [**Supplementary Figure S1**(f1, f2)], confirming excellent fluorescence responsiveness of the labeling system. Additionally, microscopic imaging of the fluorescently labeled PLA-NPs demonstrated a labeling efficiency of up to 95% (**Supplementary Figure S2**).

Building on this, the long-term stability of fluorescently labeled PLA-NPs was evaluated across two dimensions: “fluorescence stability” and “system stability.” For fluorescence stability validation, the dynamic monitoring of the fluorescence signal from fluorescently labeled PLA-NPs was conducted over one month (**Supplementary Table S1**). Although the particle fluorescence signals exhibited a certain degree of quenching, no statistically significant difference in fluorescence quenching rates was observed between the two PLA-NPs particle sizes (*p >*0.05), indicating that particle size exerts a negligible influence on the fluorescence decay rate of this labeling system. For system stability validation, a 6-month dark incubation disturbance test was performed on the PLA-NPs suspension. After 6 months, component analysis of the suspension detected no additional impurities (**Supplementary Figure S3**).

### 3.2 Translocation and uptake of PLA-NPs by pakchoi

In this study, LSCM was used to conduct qualitative observations of root tips, root bases, stems, and leaves in pakchoi seedlings across different treatment groups. Combined with semi-quantitative analysis of fluorescence intensity using ImageJ software, this approach systematically investigated the effects of PLA-NPs particle size, treatment concentration, and exposure time on the distribution and accumulation of PLA-NPs within seedlings from both qualitative and quantitative perspectives.

Under 536 nm excitation, no fluorescence signal was detected in root tip (a), root base (b), or stem (c) sections of pakchoi seedlings in the blank control group (**Supplementary Figure S4**). This confirms that the 536 nm excitation wavelength effectively eliminates autofluorescence interference from the plant itself, thereby ensuring the reliability of subsequent experimental results.

**Figure 1:**
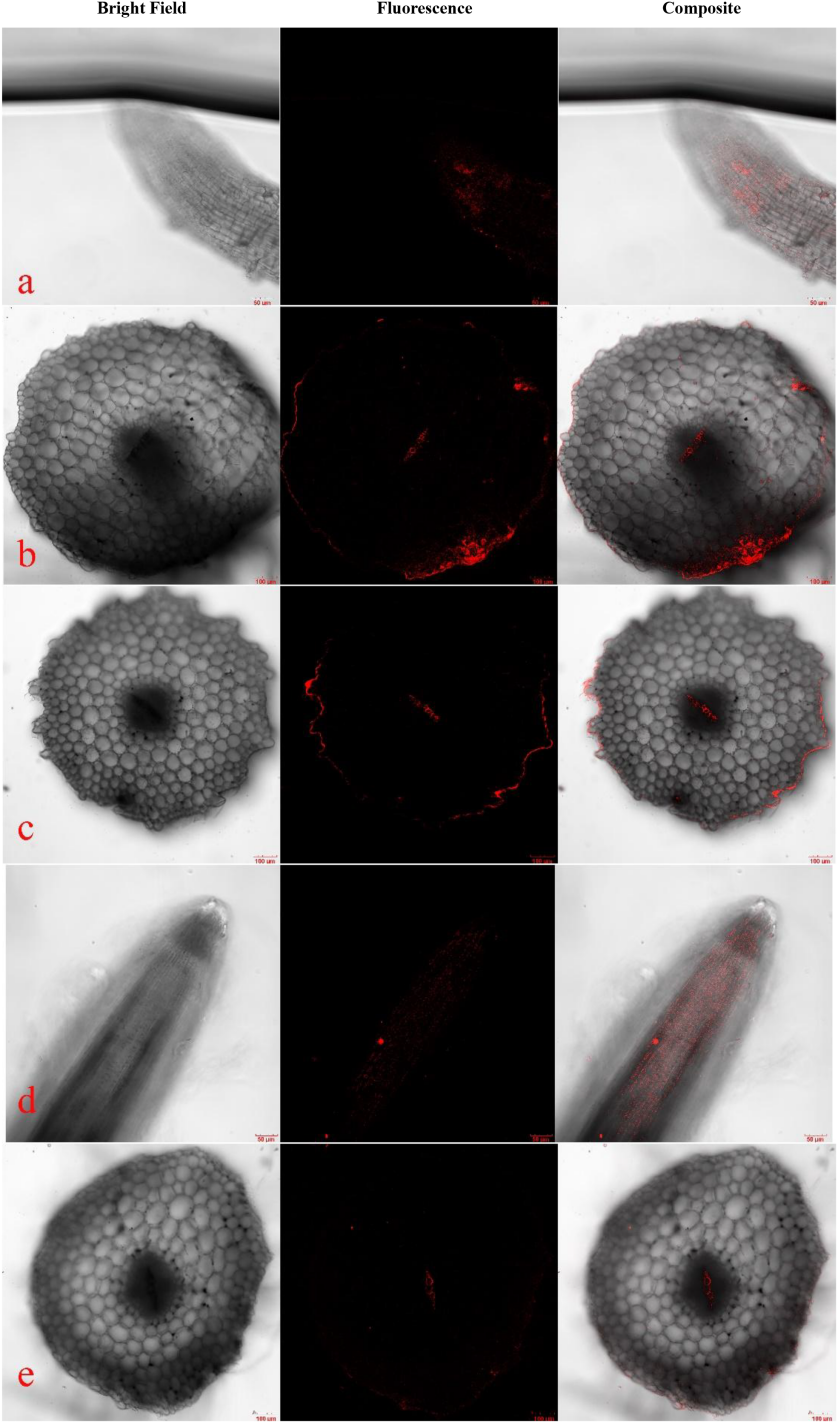
Root tips, bases, and stems of pakchoi seedlings treated with 50 mg/L PLA-NPs for seven days are shown in LSCM photographs. Sections of the root tips **(a)**, root bases **(b)**, and stems **(c)** of seedlings treated with 50 mg/L of 170 nm PLA-NPs for seven days are shown in confocal pictures **(a–c)**. Confocal pictures of portions of the root bases **(e)** and tips **(d)** of seedlings treated with 50 mg/L of 330 nm PLA-NPs for seven days are shown in **(d, e)**.

After exposing pakchoi seedlings to 50 mg/L-170 nm PLA-NPs for 7 days, red fluorescence signals were observed in both the outer epidermis of root tips and root vascular bundles (**Figure 1a**), with a corresponding fluorescence intensity of 15.3728 arbitrary units (AU)—significantly higher than that of the control group at the same time point (**Supplementary Table S2**). At the same concentration, seedlings treated with 330 nm PLA-NPs also exhibited fluorescence signals in these regions (**Figure 1d**). However, the signal intensity was lower than that in the 170 nm treatment group. Semi-quantitative results further confirmed this difference: the root tip fluorescence intensity in the 50 mg/L-330 nm group was 14.3180 AU, which was lower than the 15.3728 AU in the 50 mg/L-170 nm group (**Supplementary Table S2**), indicating that pakchoi seedling roots more readily adsorb smaller-sized PLA-NPs.

Following 7 days of exposure, fluorescence signals were also detected in root bases and stem vascular bundles—tissues with no direct contact with the culture medium. Semi-quantitative data showed that the fluorescence intensities in the root base and stem of the 20 mg/L-170 nm group were 13.6064 AU and 11.5736 AU, respectively, while those in the 20 mg/L-330 nm group were 12.9766 AU and 10.9390 AU (**Supplementary Table S2**); both groups had significantly higher intensities than the control. This confirms that both 170 nm and 330 nm PLA-NPs migrate upward in pakchoi seedlings, with the 170 nm group (**Figures 1b, c**) exhibiting markedly stronger fluorescence signals in root bases and stem vascular bundles than the 330 nm group (**Figure 1e**). Collectively, these results demonstrate that NPs absorbed by seedling roots can migrate to aboveground tissues via xylem vascular bundles. Notably, the stronger fluorescence signals in the 170 nm PLA-NPs treatment group confirm that smaller-sized PLA-NPs possess superior mobility within plant tissues.

**Figure 2:**
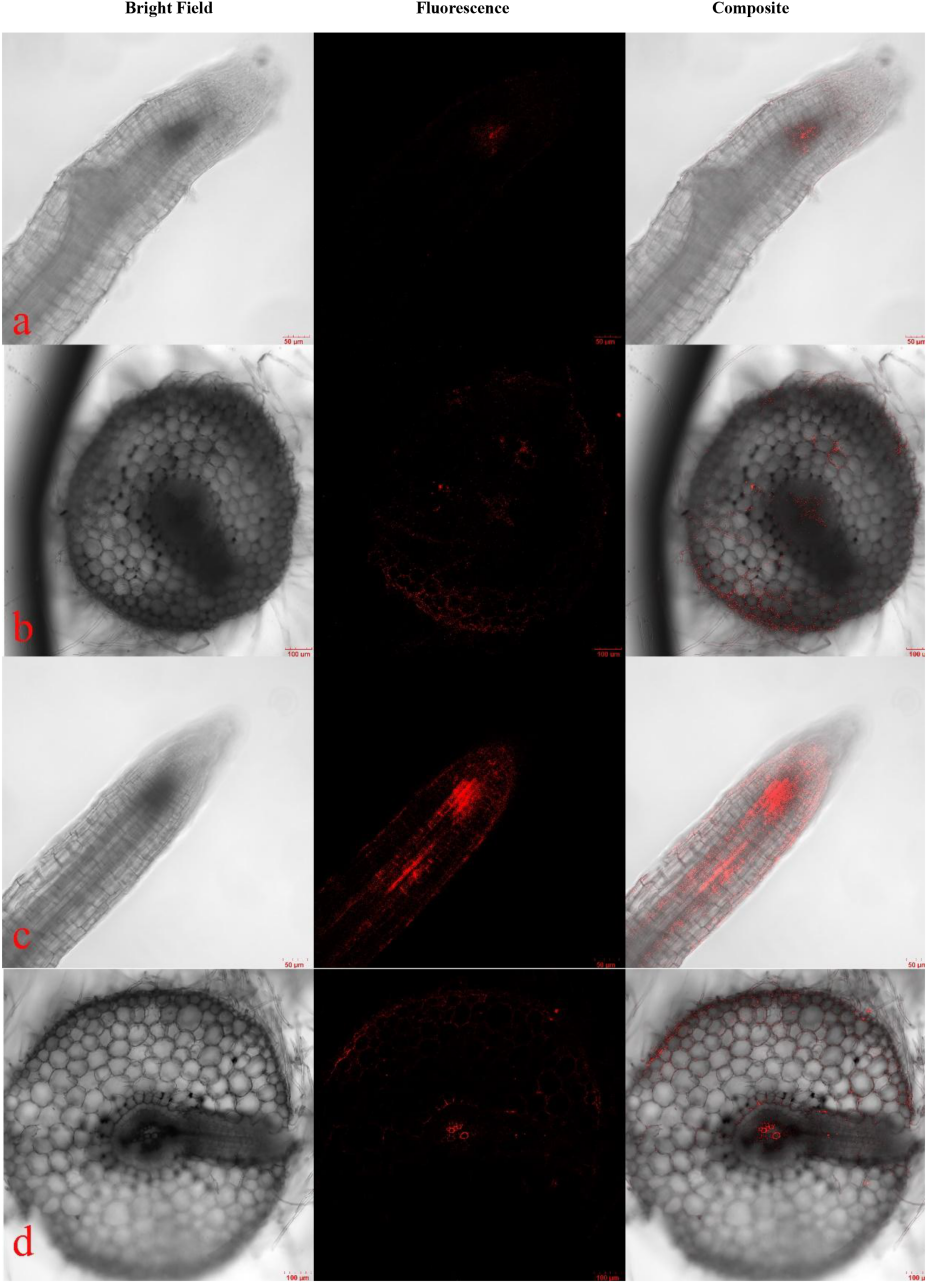
LSCM images of root tips and root bases of pakchoi seedlings treated with 170 nm PLA-NPs at concentrations of 20 mg/L **(a, b)** and 50 mg/L **(c, d)** for 14 days. **(a, b)** show microscopic images of root tips and root bases in the 20 mg/L-170 nm treatment group, while **(c, d)** show those of root tips and root bases in the 50 mg/L-170 nm treatment group.

**Figure 2** presents LSCM images of pakchoi seedling roots following 14 days of exposure to 170 nm PLA-NPs at different concentrations. Despite ultrasonic cleaning of the roots, fluorescent signals remained detectable on the root epidermis, confirming the tight adhesion of PLA-NPs to the root surface. Microscopic observation of root tips revealed PLA-NPs accumulation at the root cap. As the exposure concentration increased, fluorescence intensity gradually enhanced in the outer epidermis of the root, the apical meristem, and the vascular bundles. Semi-quantitative data showed that at 14 days of exposure and 170 nm particle size, the root tip fluorescence intensity in the 50 mg/L group (17.7906 AU) was significantly higher than that in the 20 mg/L group (15.7276 AU) (**Supplementary Table S2**)—fully corroborating the concentration-dependent enhancement of fluorescence intensity. A similar fluorescence distribution pattern was observed in stems and petiole bases (**Supplementary Figure S5**). Red fluorescence signals were detected in the vascular bundles of stems and petioles in both the 20 mg/L and 50 mg/L treatment groups; in the 50 mg/L-170 nm group, the fluorescence intensities of stems and petioles were 14.8066 AU and 11.6864 AU, respectively, both exceeding those in the 20 mg/L-170 nm group (**Supplementary Table S2**). Furthermore, fluorescence signals in petioles were significantly weaker than those in stems and roots—a finding supported by semi-quantitative data (14 days, 50 mg/L-170 nm group: root tip, 17.7906 AU; petiole, 11.6864 AU). These results indicate that PLA-NPs primarily accumulate in roots, with only limited migration to aboveground tissues.

**Figure 3:**
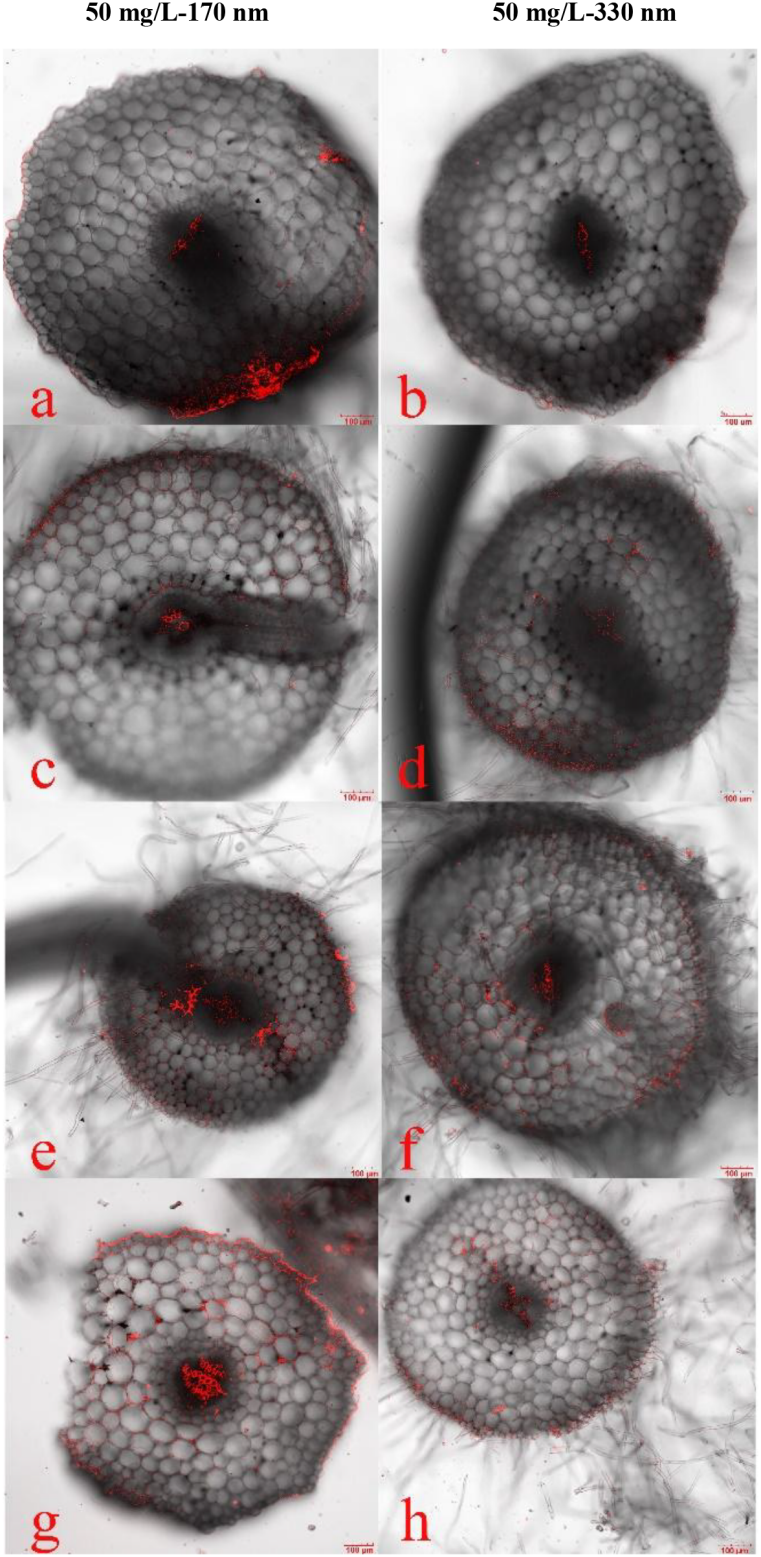
LSCM images of root bases of pakchoi seedlings exposed for different durations. In the figure, labeled groups **(a-f)**, and **(g, h)** represent microscopic images of roots exposed for 7, 14, 21, and 28 days, respectively. Among these, groups **(a, c, e, g)** correspond to the 50 mg/L-170 nm PLA-NPs treatment group, and groups **(b, d, f, h)** to the 50 mg/L-330 nm PLA-NPs treatment group.

In addition to PLA-NPs particle size and concentration, exposure time also regulates the migration and distribution of PLA-NPs in pakchoi plants. **Figure 3** presents LSCM images of the root base region of pakchoi seedlings under composite treatment, captured after different exposure durations. Results show that after 7 days of exposure, the red fluorescent signal at the root base was characterized by a small area and weak intensity. As exposure time increased, fluorescence signal intensity gradually enhanced—a trend clearly supported by semi-quantitative data. Taking root tips of the 50 mg/L-170 nm group as an example, fluorescence intensity increased from 15.3728 AU at 7 days to 19.2364 AU at 28 days; in the 20 mg/L-330 nm group, petiole fluorescence intensity increased from 9.0102 AU at 7 days to 12.1884 AU at 28 days. These results demonstrate that the uptake and accumulation of PLA-NPs by roots exhibit time-dependent characteristics, with cumulative and accelerating effects over time. This trend remains consistent regardless of variations in particle size or concentration.

**Figure 4:**
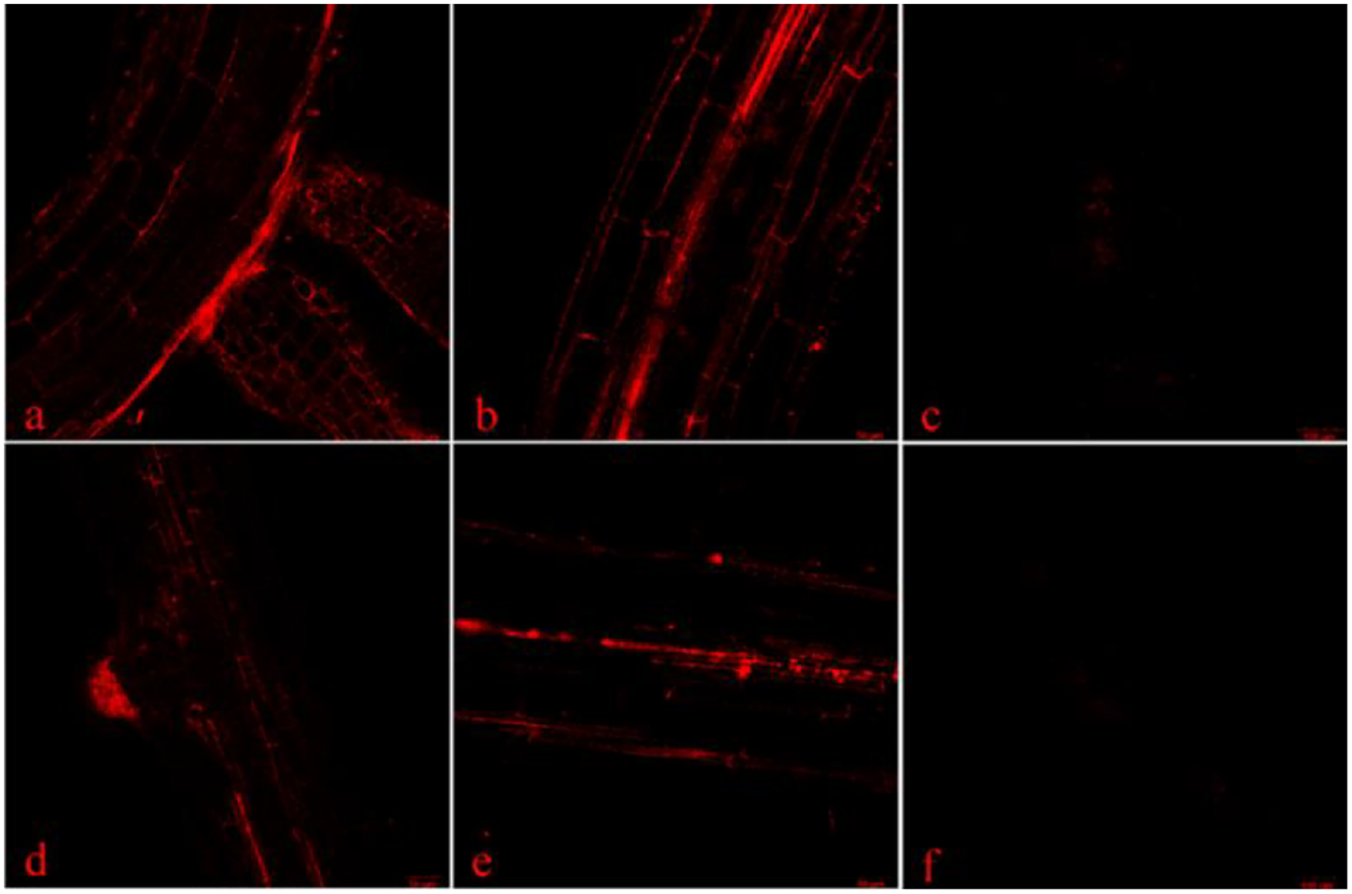
Transverse root and petiole sections and the primary root-lateral root junction of pakchoi seedlings after 14 days of treatment with 50 mg/L PLA-NPs solution at 170 nm **(a-c)** and 330 nm **(d-f)** were examined by LSCM. **(a, d)** represent the primary root-lateral root junctions; **(b, e)** are root cross-sections; **(c, f)** are petioles.

After 14 days of treatment with 50 mg/L-170 nm and 20mg/L-330 nm PLA-NPs solutions (**Figure 4**), fluorescent signals were detected at the primary root-lateral root junction (a, d), in the root pith and cortex (b, e), and within petiole vascular bundles (c, f). These signals were predominantly localized to the root apical meristem (**Figure 3e**) and primary root-lateral root junctions (**Figures 4a, d**), indicating that PLA-NPs likely enter seedling roots via the apical meristem and primary-lateral root junctions, are transported to root vascular bundles via the apoplast, and then translocate upward to the petiole base under the driving force of transpiration (**Figures 4c, f**). Furthermore, fluorescence signal intensity was higher in the 50 mg/L-170 nm treatment group than in the 50 mg/L-330 nm group.

**Figure 5:**
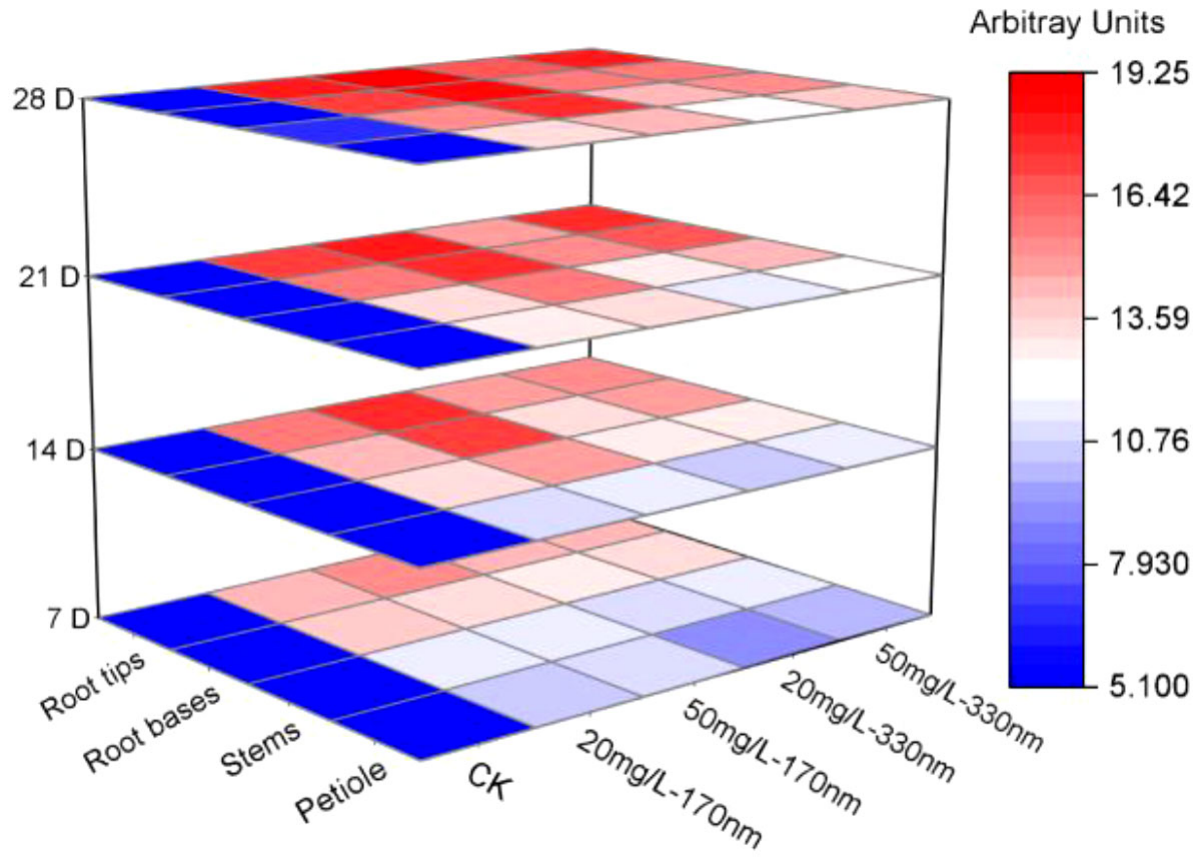
Relative fluorescence intensity in the root tip, root base, stem, and petiole of each treatment group at different time points.

In summary, at identical concentrations, fluorescence intensity exhibited a significant negative correlation with nanoparticle size; smaller particles resulted in higher fluorescence intensity in root tips, root bases, stems, and petioles. At a fixed particle size, fluorescence intensity showed a clear positive correlation with treatment concentration. As concentration increased, fluorescence intensity rose across all regions; although roots and petioles displayed slight differences in sensitivity to concentration changes, concentration-dependent enhancement remained the dominant trend overall. Across all treatment groups, fluorescence intensity at all sites increased continuously with extended culture duration. This time-dependent effect exhibited cumulative enhancement, with the accumulation of fluorescent signals accelerating over time—a trend unaffected by other variables (**Figure 5**; **Supplementary Table S2**).

### 3.3 Physiological indicators of pakchoi seedlings

#### 3.3.1 Growth indicators of pakchoi

The basic indicators and growth status of pakchoi seedlings across different treatment groups are presented in **Supplementary Table S3**; **Supplementary Figure S6**. Germination rates showed no significant difference between the control and treatment groups (*p >*0.05), indicating that seeds germinated normally. At equivalent concentrations, biomass increased with PLA-NPs particle size. The 50 mg/L-330 nm PLA-NPs group showed a significant increase in plant height of pakchoi plants (*p<*0.05), with a 25.58% increase, while other treatments exhibited no significant difference from the control. There was a significant difference (*p<*0.05) in the biomass of pakchoi between the PLA-NPs treatments and the control. The biomass of the 170 nm PLA-NPs treatment group increased by 31.37% and 37.25%, and that of the 330 nm treatment group increased by 52.94% and 56.86% under the concentrations of 20 and 50 mg/L, respectively. Biomass promotion increased with PLA-NPs concentration, with significantly lower values in the 20 mg/L group compared to the 50 mg/L group. There were no appreciable variations between the four treatment groups and the control, and root length trended similarly to plant height (*p >*0.05).

#### 3.3.2 Photosynthesis in pakchoi

**Figure 6:**
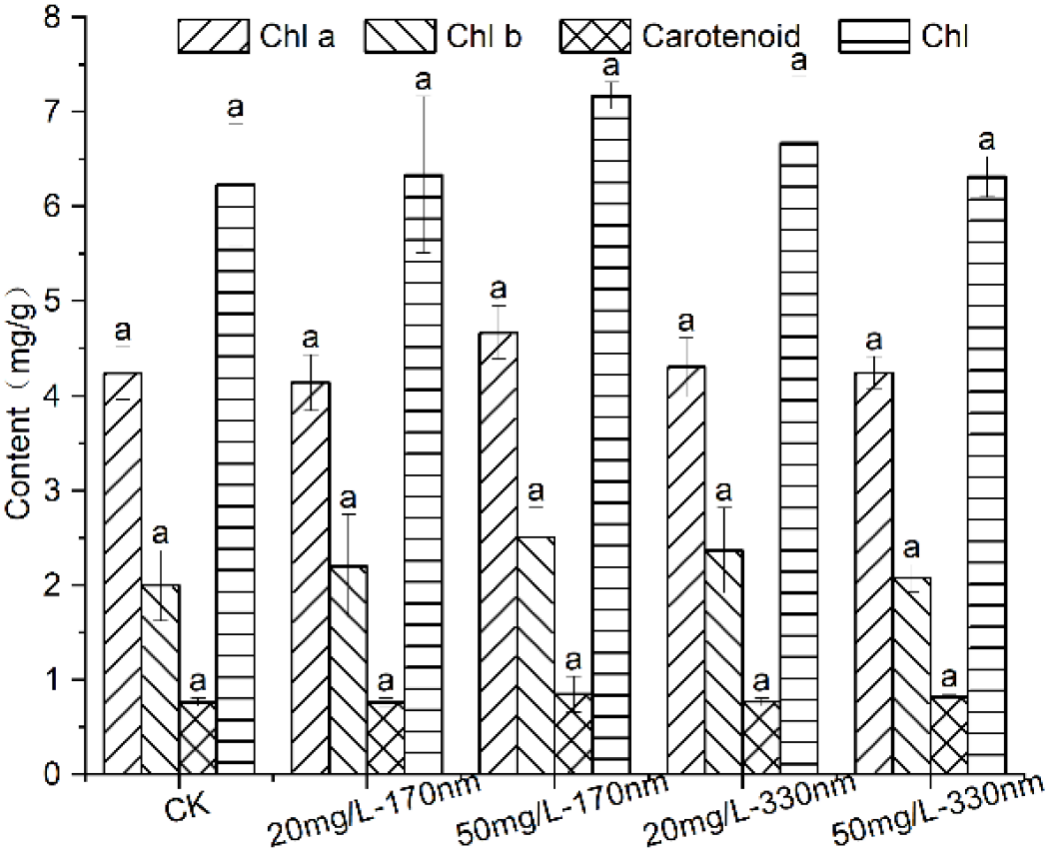
Changes in pigment content of pakchoi seedlings exposed to different treatment groups. Different letters on the bars indicate significant differences according to Duncan’s test (*p >*0.05).

Pigment contents in response to PLA-NPs of two particle sizes at different concentrations are presented in **Figure 6**. All treatment groups had higher levels of carotenoids, total chlorophyll, chlorophyll a, and chlorophyll b overall than the control, but no statistically significant changes were found (*p >*0.05). While the 330 nm group displayed the opposite tendency, with higher pigment concentrations in the 20 mg/L treatment compared to the 50 mg/L treatment, the 170 nm group showed higher pigment contents in the 50 mg/L treatment than in the 20 mg/L treatment. Specifically, compared to the control, the 50 mg/L-170 nm PLA-NPs treatment increased chlorophyll a, chlorophyll b, carotenoids, and total chlorophyll by 10.18%, 25.43%, 11.18%, and 15.07%, respectively, while the 20 mg/L-330 nm treatment increased these pigments by 1.56%, 18.61%, 0.81%, and 7.02%, respectively. These results indicate that the 50 mg/L-170 nm treatment exerted a relatively more substantial promotional effect on pigment contents in pakchoi seedlings.

#### 3.3.3 Antioxidant defense system of pakchoi

**Figure 7:**
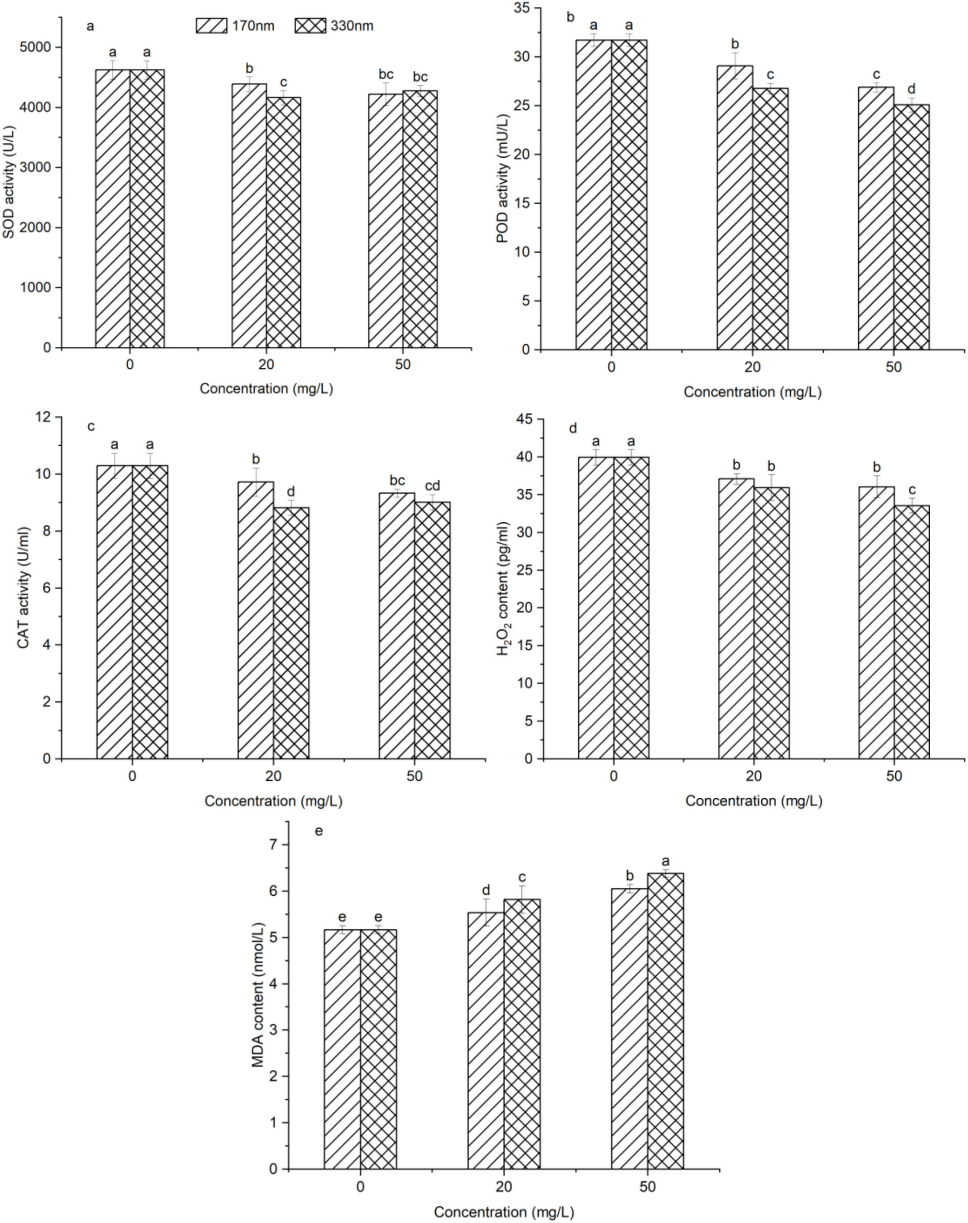
Effects of different treatment groups on the antioxidant defense system in pakchoi. Different letters on the bars indicate significant differences according to Duncan’s test (*p<*0.05).

**Figure 7** presents the effects of different treatment groups on the antioxidant defense system of pakchoi. From (a)-(c) show that all treatment groups showed a significant decrease in SOD, POD, and CAT activities as compared to the control (*p* < 0.05), indicating that PLA-NPs induce reactive oxygen species (ROS) accumulation and increased oxidative stress levels in pakchoi seedlings, which in turn cause cellular and tissue damage and disrupt normal cellular physiological functions. Correlation analysis revealed a significant correlation between SOD, POD, and CAT activities and PLA-NPs particle size (*p* < 0.05): The antioxidant system of pakchoi seedlings was more strongly inhibited by 330 nm PLA-NPs, which decreased SOD, POD, and CAT activities by 9.97%, 15.62%, and 14.32% at 20 mg/L and 7.52%, 20.32%, and 12.48% at 50 mg/L, respectively. The H_2_O_2_ content trend mirrored the SOD activity trend (**Figure 7d**). MDA is frequently used as a measure of oxidative stress intensity. Higher levels of MDA, a byproduct of lipid peroxidation, indicate more severe oxidative damage to cell membranes. As shown in **Figure 7e**, MDA contents in the 20 mg/L-170 nm, 50 mg/L-170 nm, 20 mg/L-330 nm, and 50 mg/L-330 nm treatment groups increased by 7.06%, 17.12%, 12.56%, and 23.40%, respectively, confirming that PLA-NPs cause significant oxidative damage to pakchoi seedlings.

#### 3.3.4 Osmoregulatory systems in pakchoi

**Figure 8:**
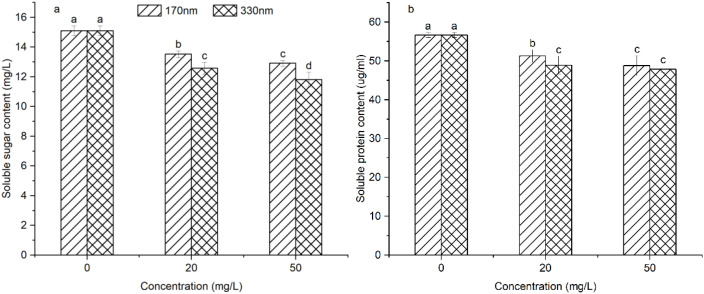
Effects of different treatment groups on the osmoregulatory system of pakchoi. Different letters on the bars indicate significant differences according to Duncan’s test (*p* < 0.05).

Soluble sugars and soluble proteins are key osmoregulatory substances in plants, and changes in their content reflect the plant’s adaptive capacity to environmental conditions. The levels of both drugs were significantly lower in all treatment groups than in the control group (*p<*0.05), as shown in **Figures 8a, b**; at equivalent concentrations, larger particle sizes exerted more potent inhibitory effects on both, for 330 nm PLA-NPs, soluble sugar and soluble protein contents in the 20 mg/L treatment group were reduced by 16.73% and 13.70% relative to the control, respectively, while reductions of 21.76% and 15.57% were observed in the 50 mg/L treatment group. The results demonstrate that exposure to PLA-NPs reduces the osmoregulatory capacity of pakchoi, which in turn impairs its ability to uptake water and nutrients.

## 4 Discussion

### 4.1 Mechanisms of translocation and uptake of PLA-NPs by pakchoi

LSCM revealed that PLA-NPs were widely distributed in the vascular systems of roots, stems, and petioles of pakchoi, with primary enrichment in the root stele and stem vascular bundles. At equivalent concentrations, the fluorescence intensity of 170 nm PLA-NPs was significantly higher than that of 330 nm PLA-NPs, consistent with findings by Li et al. (2019) that small-sized particles are more readily absorbed and transported in lettuce and cucumber. This phenomenon is related not only to the smaller size of PLA-NPs, which facilitates penetration of root cell interstices and cell wall pores (Li et al., 2019), but also to their surface charge density and adsorption properties, which contribute significantly (Zhao et al., 2024). The larger specific surface area and relatively denser surface charge distribution of small-sized PLA-NPs enhance their adsorption and internalization on root surfaces via stronger binding to root exudates (Dong et al., 2021). Additionally, as a degradable polyester, PLA may undergo esterase-mediated enzymatic degradation in plants (Lee et al., 2014). It has been reported that PLA is gradually hydrolyzed, with ester bond cleavage producing smaller fragments, under the action of esterases secreted by soil microorganisms and plant roots (Shalem et al., 2024); this process may further enhance its mobility in the vascular system. In contrast, traditional non-degradable polymers tend to accumulate stably due to their high hydrophobicity and chemical stability, making similar degradation in plants less likely.

The fluorescence signal intensity at the root base was much higher in the 50 mg/L treatment group than in the 20 mg/L group at equivalent particle sizes. This could be related to the nanoparticles’ surface charge-mediated adsorption capabilities. PLA-NPs carry a weak negative charge in aqueous solutions due to oxygen-containing functional groups (**Supplementary Figure S1**(c)). Root epidermal cell walls are rich in negatively charged polysaccharides such as pectin (Parkinson et al., 2022). When PLA-NPs approach the root epidermis, solution cations (e.g., Ca^2+^, Mg^2+^) can act as bridges to mediate the adsorption of PLA-NPs to the root epidermis via electrostatic attraction (Allen et al., 2019). Conversely, increased collision frequency between PLA-NPs at high concentrations facilitates aggregation into larger clusters, expanding the contact area with the root epidermis and thereby enhancing adsorption (Ouyang et al., 2024). This aligns with the concentration-dependent accumulation pattern of PS-NPs reported by Lian et al. (2021). Additionally, studies on styrene maleic anhydride (SMA) nanoparticles have demonstrated that plant accumulation of NPs is linearly correlated with exposure concentration (Zhang et al., 2019), further confirming that concentration is a key factor regulating uptake.

Available results indicate that PLA-NPs fluorescence signals are primarily concentrated in root vascular bundles and cortical tissues, leading to the hypothesis that their transport occurs mainly via the plasmalemma pathway (Liu et al., 2022). Due to active cell division in the apical meristem of young roots and incomplete development of the Casparian strip, PLA-NPs can migrate to the vascular system through cell wall interstices (Wang et al., 2022). Additionally, natural clefts at the primary root-lateral root junction provide an alternative entry route for PLA-NPs, consistent with both the cleft structures observed in sections and previous conclusions that “the lateral root initiation zone serves as a shortcut for nanoparticle entry” (Li et al., 2022, Li et al., 2020). For the migration of large-sized PLA-NPs, in addition to relying on cleavage and exosome pathways, their migration may be associated with their mechanical elasticity and the dynamic response of plant cell walls (Liu et al., 2024). PLA, which has a lower Young’s modulus than plant cell walls, may deform under shear stress and pass through cell walls with smaller pores during transport, or induce localized cell wall distortion to form temporary channels (Ouyang et al., 2024). Additionally, endocytosis may be involved in the internalization of PLA-NPs (Jiang et al., 2022). It has been demonstrated that positively charged nanoparticles are more likely to enter cells via endocytosis due to stronger electrostatic attraction to the cell membrane (Shi et al., 2024). Although PLA-NPs are negatively charged, in the complex environment of the plant rhizosphere, they may bind to cations in root secretions or undergo surface functional group modification—processes that can alter their surface charge properties—allowing recognition and endocytosis by the cell membrane (Zhao et al., 2024).

Once in the vascular system, PLA-NPs are transported along vascular bundles via water and nutrient flow driven by transpiration pull, a mechanism similar to that of non-degradable polymers (Luo et al., 2022). However, the degradability of PLA distinguishes it fundamentally from non-degradable polymers (Sun et al., 2022). For example, due to their non-degradable nature, PS-NPs accumulate persistently in plants. In contrast, PLA may be gradually degraded into lactate monomers by esterases, with these monomers partially utilized in plant metabolism, resulting in a potentially weaker long-term accumulation effect than PS-NPs. Additionally, PLA exhibits higher hydrophilicity than PS (Zhao et al., 2024), resulting in stronger interactions with root secretions compared to hydrophobic PS-NPs. This enhanced hydrophilicity may facilitate the transport of PLA-NPs via the exosomal pathway, whereas PS-NPs rely more on endocytosis.

### 4.2 Effects of PLA-NPs on physiological indicators of pakchoi seedlings

NPs accumulation in plants can induce toxic effects, thereby affecting plant growth and development. Seed germination, a key stage in the plant life cycle, reflects a seed’s ability to adapt to adverse environments. Guo et al. (2022) observed that the germination rates of T. repens and I. balsamina decreased significantly with increasing PS concentration, whereas no significant change was observed in O. violaceus seeds. Li et al. (2024) noted that the consequences of PS on pea seed germination rely on particle size and concentration: small-sized, high-concentration PS exerted the most potent inhibitory effect, while low-concentration or large-sized PS had a promotional effect. In the present study, however, exposure to different concentrations of PLA-NPs did not significantly alter the germination rate of pakchoi seedlings. This disparity can be attributed to variations in the physiological traits and growth patterns of different plant species, which result in varying sensitivities and tolerances to MPs.

Photosynthetic pigments drive plant growth by absorbing and converting light energy into chemical energy. Previous studies have shown that MPs typically inhibit plant photosynthetic rates and reduce chlorophyll content, thereby hindering nutrient accumulation and suppressing crop growth (Jia et al., 2025). In the present study, however, PLA-NPs increased chlorophyll content in pakchoi, promoted leaf growth and expansion of the photosynthetic area, and ultimately enhanced biomass. This may be attributed to PLA-NPs’ adsorption on root surfaces and their *in vivo* accumulation, which could upregulate the expression of genes related to chloroplast development (Zhang et al., 2017); alternatively, PLA-NPs may accelerate chlorophyll synthesis by promoting root uptake of minerals and increasing chlorophyllase activity (Zheng et al., 2005). These findings suggest that the impacts of NPs on plant photosynthetic systems may vary based on plant species and the intrinsic properties of the plastic.

The antioxidant defense system and osmoregulatory system are core mechanisms by which plants cope with environmental stresses, playing a critical role in maintaining cellular function and stability. In the current investigation, PLA-NPs considerably decreased the activities of SOD, POD, and CAT in pakchoi, indicating that the oxidative stress induced by PLA-NPs exceeded the tolerance range of the antioxidant system, leading to its dysfunction. However, Zhang et al. (2023a) found that at high chromium concentrations, polyamide MPs and PLA-MPs markedly enhanced SOD, POD, and CAT activity in plants, indicating more robust antioxidative stress responses and reflecting stronger antioxidative stress responses. Adamczyk et al. (2024) also noted that low concentrations of PBAT MPs increased SOD and CAT activities, with elevated MDA levels only observed at high concentrations. These discrepancies may be associated with the chemical properties, particle size, and surface characteristics of MPs, as well as the plant species. Additionally, PLA-NPs treatment decreased soluble sugar and soluble protein contents in pakchoi in this study. In contrast, Zhang et al. (2023b) demonstrated that PS-MPs significantly increased the levels of these two substances in strawberry seedlings, hypothesizing that PS-MPs induce osmotic stress, prompting plants to accumulate osmoregulatory substances to maintain cellular osmotic balance. The reduction in osmoregulators observed herein may indicate that pakchoi did not experience significant osmotic stress due to PLA-NPs, or that its capacity to upregulate these substances for stress coping is limited.

In this study, the observations that PLA-NPs increases plant biomass and pigment content while decreasing antioxidant enzyme activity and osmotic regulation capacity are not contradictory. Instead, these findings reflect its concentration-dependent dual regulatory effects on plants and the plant’s “growth-priority” resource allocation strategy. Regarding growth promotion, PLA—as a bio-based material—can be converted into the endogenous auxin phenylacetic acid to regulate plant growth (Maki et al., 2022). By increasing chlorophyll content, PLA-NPs enhances photosynthetic efficiency, thereby laying the foundation for biomass accumulation. Increases in biomass and plant height, as core growth indicators, directly corroborate the subsequent regulatory mechanisms at the physiological and metabolic levels, further supporting the validity of the “growth-first” strategy. At the stress response level, reduced activities of SOD, POD, CAT, and H_2_O_2_ content indicate that PLA-NPs does not induce significant oxidative stress but rather alleviates basal oxidative stress. This enables plants to conserve metabolic costs by avoiding the need to maintain high antioxidant enzyme activity, consistent with the findings of (Shi et al., 2018), who reported that “low-concentration PLA derivatives reduce wheat POD activity while improving growth indicators.” The increase in MDA content alongside decreases in soluble sugars and soluble proteins reflects a growth-metabolism trade-off strategy: PLA-NPs induced photosynthetic enhancement and rapid biomass accumulation prioritize carbon and nitrogen allocation toward growth processes rather than osmoregulatory substance synthesis or membrane damage repair, leading to temporary MDA accumulation. This aligns with the findings of (Gao et al., 2024), who observed “a trade-off pattern where growth-promoting substances prioritize carbon allocation toward growth.” In summary, the available physiological data exhibit inherent logical consistency, clearly defining PLA-NPs’s “growth-priority” regulatory model—characterized by enhanced biomass accumulation via photosynthetic promotion, coupled with downregulated non-essential antioxidant and osmotic regulation metabolism through optimized resource allocation.

## 5 Conclusion

For the first time, this study demonstrates that pakchoi roots can take up BPs, accumulate in plants, and migrate to aboveground tissues, with small-sized PLA-NPs showing a greater propensity for accumulation in pakchoi and upward migration. LSCM observations revealed that PLA-NPs fluorescence signals were primarily concentrated in the stele and vascular bundles, indicating migration to aboveground tissues via exosomal transport. Physiological and biochemical analyses revealed that the uptake and accumulation of PLA-NPs affected the synthesis of photosynthetic pigments, the antioxidant defense system, and the osmoregulatory system in pakchoi seedlings. Specifically, 330 nm PLA-NPs exerted more pronounced effects, and concentration was an additional key factor: the 50 mg/L treatment had a more significant impact compared to the 20 mg/L treatment. It should be noted that this study was conducted under hydroponic conditions, where plants could directly contact PLA-NPs, potentially leading to overestimation of uptake. Furthermore, the study did not further explore the applicability and generalizability of these results to soil-grown plants, representing a significant limitation. This study investigates the effects of degradable NPs on plants, which not only aids in assessing their environmental safety but also enhances understanding of their environmental behavior and potential risks.

## Data Availability

The original contributions presented in the study are included in the article/**Supplementary Material**. Further inquiries can be directed to the corresponding author.
