# Supplementary material for: Migration pattern and biochemical response characteristics of polylactic acid nanoparticles in pakchoi (Brassica chinensis L. cv. SuZhou) seedlings
Source: Front Plant Sci. 2026 Jan 30;17:1718625. doi: 10.3389/fpls.2026.1718625 (PMC12900762; doi:10.3389/fpls.2026.1718625)
Supplement: Supplementary file 1 [file DataSheet1.pdf]

# Migration pattern and biochemical response characteristics of polylactic acid nanoparticles in pakchoi (*Brassica chinensis* L. cv. SuZhou) seedlings

Xinye Zhao[1], Qing Luo[1]*, Wenju Dai[1], Yongyao Deng[1], Ning Yang[2], Xu Zhu[1], Yixuan Zheng[1], Ying Li[1, 3], Liangshan Feng[3]

[1] Key Laboratory of Ecological Restoration of Regional Contaminated Environment, Ministry of Education, College of Environment, Shenyang University, Shenyang 110044, China

[2] Plant Protection College, Shenyang Agricultural University, Shenyang 110866, China

[3] Liaoning Academy of Agricultural Sciences, Shenyang 110161, China

* Corresponding Author: luoqing@syu.edu.cn; luoqingyt@126.com

**Fig. S1** The SEM images of 170 nm (a1) and 330 nm (a2) PLA-NPs. The particle size distribution plots of 170 nm (b1) and 330 nm (b2) PLA-NPs. Zeta potential of PLA-NPs (c). The FTIR spectra of PLA-NPs (d). The excitation/emission wavelengths at 536/608 nm of Nile Red-labeled PLA-NPs (e). The LSCM of fluorescently labeled PLA-NPs at 170 nm (f1) and 330 nm (f2).

**Fig. S2** Images of 170 nm and 330 nm PLA-NPs under brightfield and fluorescence microscopy, where a and b correspond to 170 nm, and c and d correspond to 330 nm (indicating fluorescence labeling rate).

**Fig. S3** After six months of constant darkroom storage, PLA-NPs suspensions exhibited stable light absorption properties.

**Fig. S4** Cross-sections of pakchoi seedlings' root tips (a), bases (b), and stems (c) taken using LSCM without the application of PLA-NPs.

**Fig. S5** 170 nm, LSCM of stems and petiole bases of pakchoi seedlings after 14 d treatment with 20 mg/L (a-b) and 50 mg/L (c-d) PLA-NPs.

**Fig. S6** Phenotypic diagrams for different treatment groups.

**Table S1** Relative fluorescence intensity of PLA-NPs at different times.

**Table S2** Relative fluorescence intensity for each treatment group (Arbitrary Units).

**Table S3** Effect of exposure to different treatment groups on the growth of pakchoi seedlings.

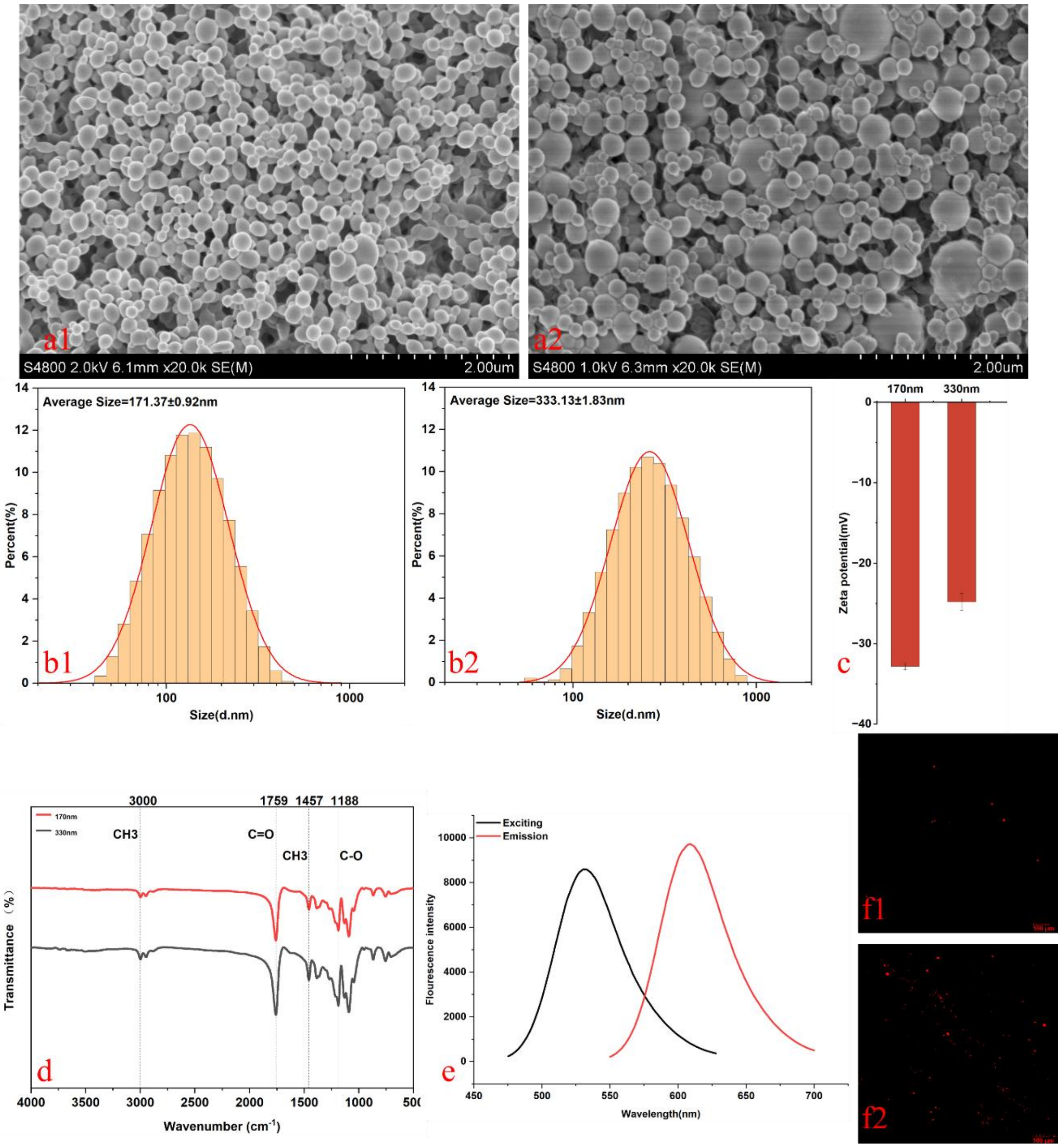


Fig. S1 The SEM images of 170 nm (a1) and 330 nm (a2) PLA-NPs. The particle size distribution plots of 170 nm (b1) and 330 nm (b2) PLA-NPs. Zeta potential of PLA-NPs (c). The FTIR spectra of PLA-NPs (d). The excitation/emission wavelengths at 536/608 nm of Nile Red-labeled PLA-NPs (e). The LSCM of fluorescently labeled PLA-NPs at 170 nm (f1) and 330 nm (f2).

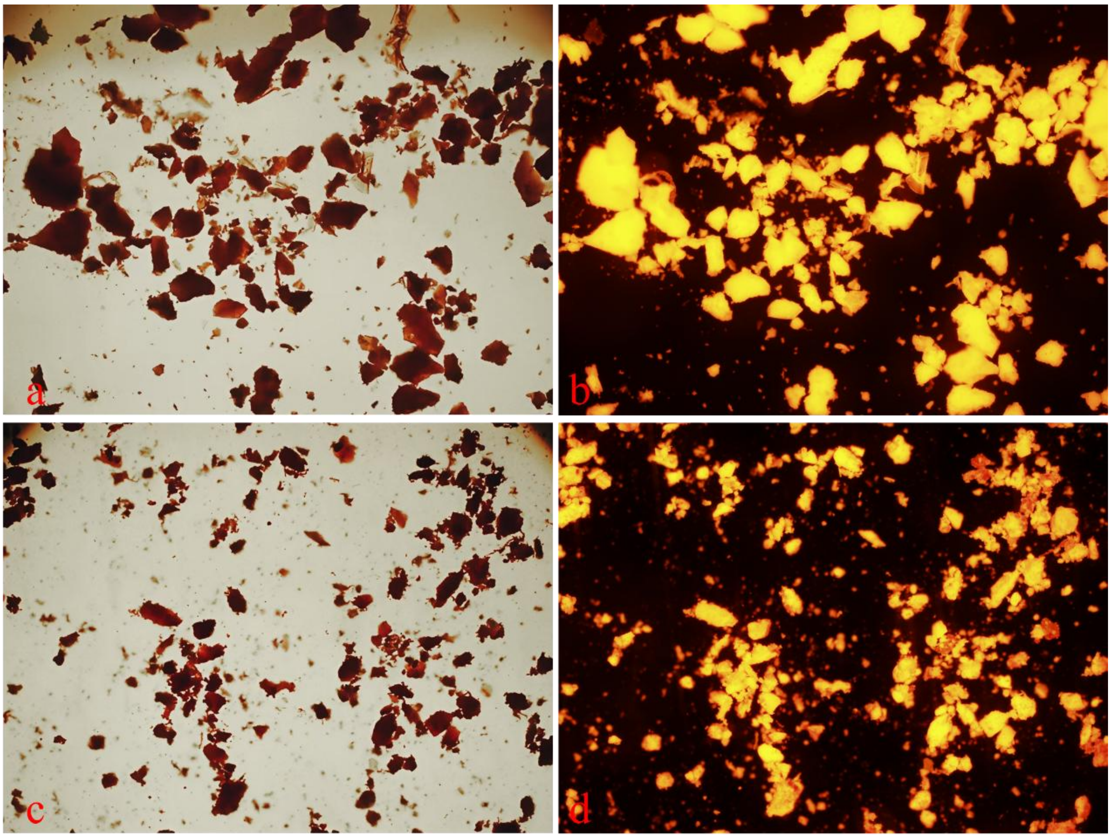


Fig. S2 Images of 170 nm and 330 nm PLA-NPs under brightfield and fluorescence microscopy, where a and b correspond to 170 nm, and c and d correspond to 330 nm. (Indicating fluorescence labeling rate).

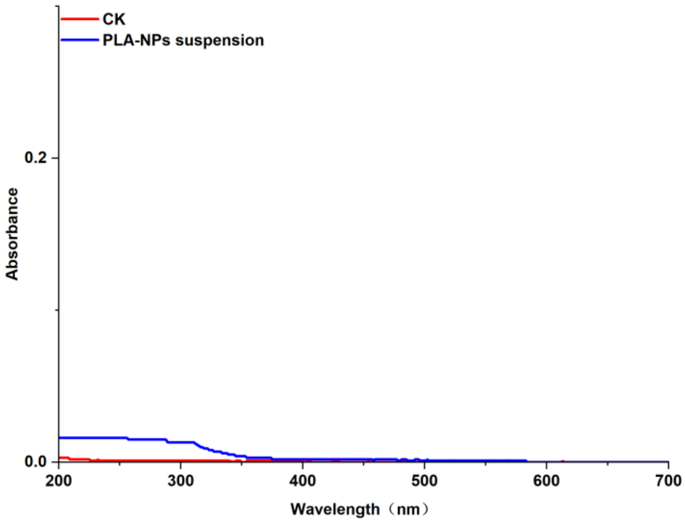


Fig. S3 After six months of constant darkroom storage, PLA-NPs suspensions exhibited stable light absorption properties.

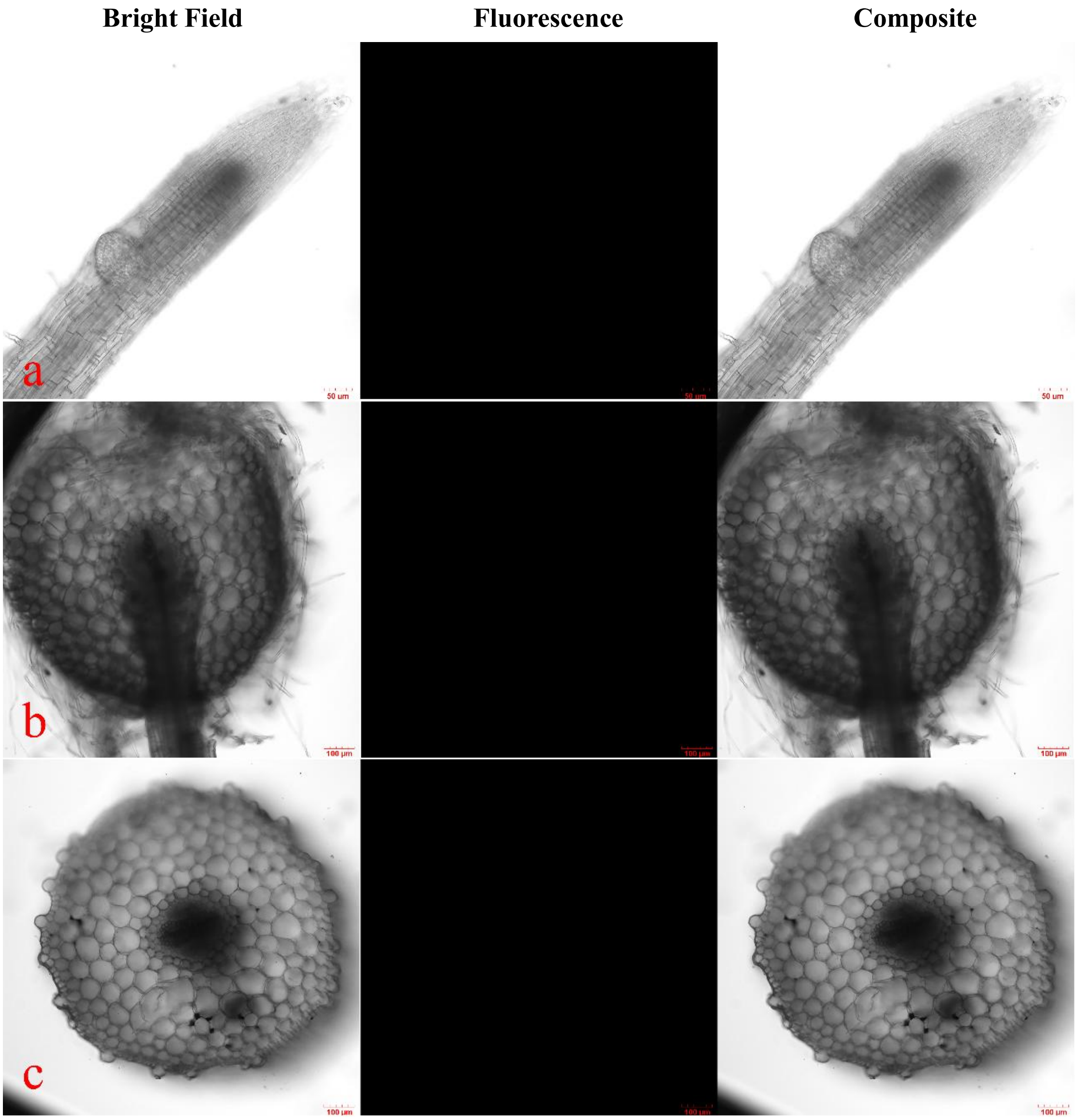


Fig. S4 Cross-sections of pakchoi seedlings' root tips (a), bases (b), and stems (c) taken using LSCM without the application of PLA-NPs.

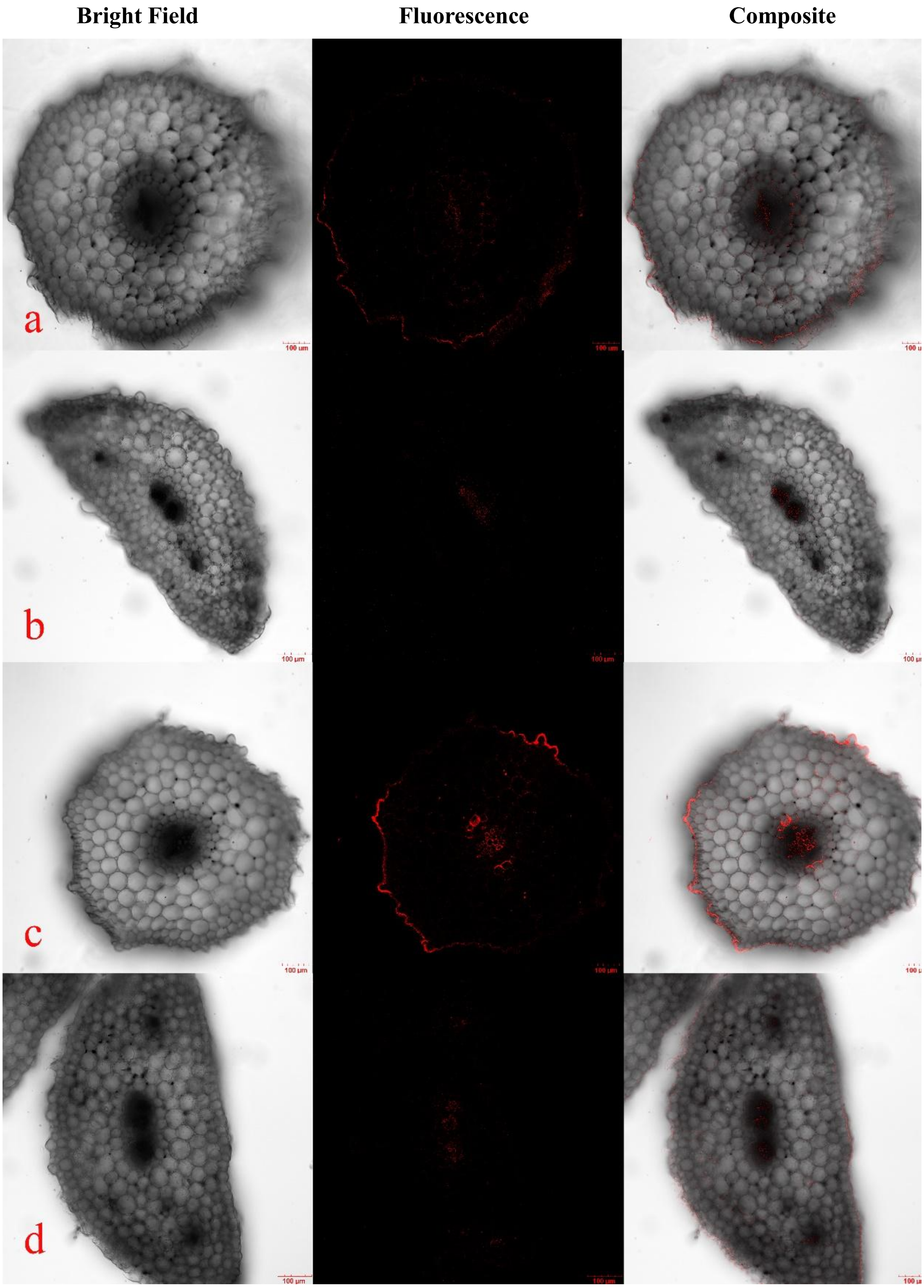

Fig. S5 170 nm, LSCM of stems and petiole bases of pakchoi seedlings after 14 d treatment with 20 mg/L (a-b) and 50 mg/L (c-d) PLA-NPs.

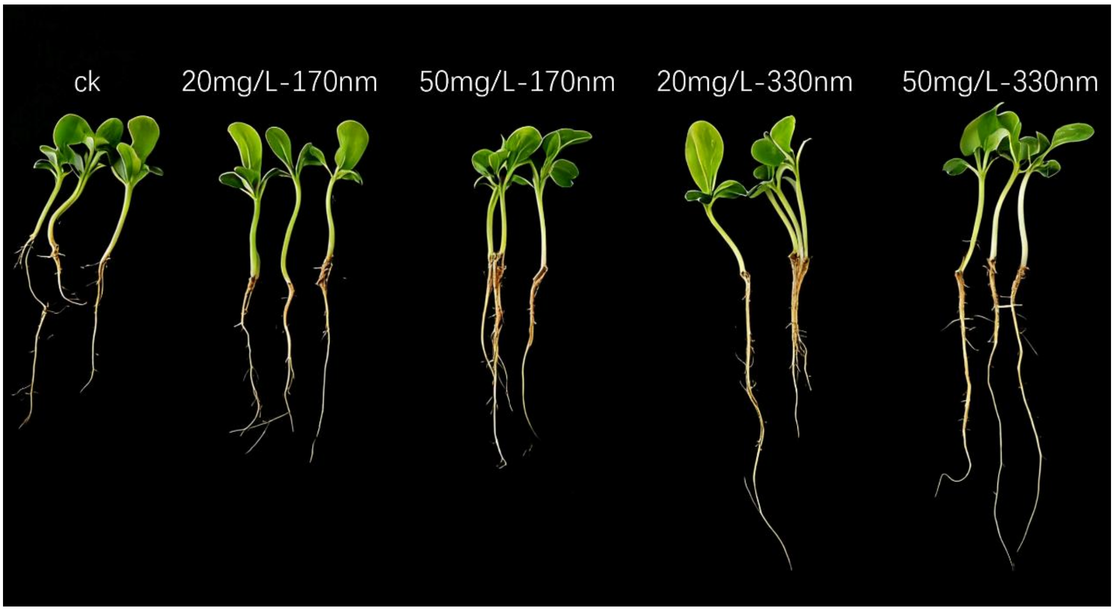


Fig. S6 Phenotypic diagrams for different treatment groups.

Table S1 Relative fluorescence intensity of PLA-NPs at different times

| Size | 0 d | 5 d | 10 d | 15 d | 20 d | 25 d | 30 d |
|---|---|---|---|---|---|---|---|
| 170nm | 128.60±9.72a | 124.84±12.47ab | 122.07±10.25ab | 117.534±10.80b | 103.57±5.36c | 100.02±12.12c | 99.09±4.50c |
| 330nm | 127.56±5.38a | 127.02±7.06a | 121.46±11.07ab | 112.87±11.19b | 98.53±8.52c | 97.61±27.02c | 92.44±12.57c |
| $F_{\text{Grop}}$, $P_{\text{Grop}}$ | | | | 1.302, 0.271 | | | |
| $F_{\text{Time}}$, $P_{\text{Time}}$ | | | | 203.397, <0.001 | | | |
| $F_{\text{Interaction}}$, $P_{\text{Interaction}}$ | | | | 0.309, 0.931 | | | |

Note: the data in the table represent means ± SDs, and significant differences between treatment groups at the 0.05 level are indicated by different lowercase letters.

Table S2 Relative fluorescence intensity for each treatment group (Arbitrary Units)

| Time | Different treatment groups | Root tips | Root bases | Stems | Petioles |
|---|---|---|---|---|---|
| 7 | CK | 5.4684±0.0178a | 5.4622±0.0267a | 5.5382±0.0347a | 5.4726±0.0437a |
| | 20mg/L-170nm | 14.2918±7.0304b | 13.6064±0.1460b | 11.5736±1.1515b | 10.2964±0.5911b |
| | 50mg/L-170nm | 15.3728±1.3603b | 13.3907±~~11.4608~~3.3133b | 11.6742±0.7744b | 10.7740±0.3586b |
| | 20mg/L-330nm | 14.2080±1.6136b | 12.9766±0.1529b | 10.9390±0.7098b | 9.0102±3.9977b |
| | 50mg/L-330nm | 14.3180±0.9776b | 13.5524±1.0370b | 11.4510±0.7852b | 10.1368±0.1796b |
| 14 | CK | 5.2858±0.1280a | 5.2858±0.6390a | 5.1284±0.1733a | 5.1166±0.1313a |
| | 20mg/L-170nm | 15.6276±4.9225b | 14.4472±0.40491b | 13.5674±0.4746b | 11.0366±0.2077b |
| | 50mg/L-170nm | 17.7906±4.2496b | 16.9006±2.3406b | 14.8066±0.5884b | 11.6864±0.7771bc |
| | 20mg/L-330nm | 14.8228±2.3516b | 13.4618±0.8256b | 12.9574±0.8439b | 10.5568±0.4098cd |
| | 50mg/L-330nm | 15.1174±0.7064b | 14.7596±0.6455c | 13.0316±0.1119c | 11.2408±0.24107d |
| 21 | CK | 5.3390±0.3220a | 5.3566±0.6139a | 5.5556±0.0888a | 5.4456±0.0372a |
| | 20mg/L-170nm | 16.9234±1.4375b | 15.8000±1.4256b | 13.4400±0.5420b | 12.9132±0.1404b |
| | 50mg/L-170nm | 18.0252±1.0649c | 17.3888±0.60248bc | 15.4912±0.2914b | 13.5800±1.6784bc |
| | 20mg/L-330nm | 14.7860±0.8146c | 15.3968±0.9170cd | 13.0704±0.3327c | 11.6546±0.8492bc |
| | 50mg/L-330nm | 17.4514±0.2647c | 16.7658±0.8675d | 14.3558±0.50175d | 12.4886±1.0334c |
| 28 | CK | 5.1592±0.1957a | 5.1764±0.2711a | 6.7418±2.1404a | 5.1118±0.0499a |
| | 20mg/L-170nm | 18.2718±0.3538b | 16.9562±0.4220b | 15.2982±1.3374b | 13.1528±0.2089b |
| | 50mg/L-170nm | 19.2364±0.5968b | 18.3698±0.5255b | 17.5930±1.5080b | 14.2014±0.7498bc |
| | 20mg/L-330nm | 16.6782±6.0994b | 15.8736±1.1513c | 14.1052±1.0005b | 12.1884±1.6172c |
| | 50mg/L-330nm | 17.9788±1.7582b | 15.8006±0.3595d | 15.1672±1.9695c | 13.7154±0.3227c |

Note: the data in the table represent means ± SDs (n=3)~~,~~ and significant differences between treatment groups at the 0.05 level are indicated by different lowercase

letters.

Table S3 Effect of exposure to different treatment groups on the growth of pakchoi seedlings.

| Different treatment groups | Germination rate/% | Biomass/g | Plant height/mm | Root length/mm |
|---|---|---|---|---|
| CK | 95±3.54ab | 0.51±0.09d | 41.67±2.89b | 68.67±6.11a |
| 20mg/L-170nm | 95±6.12ab | 0.67±0.03c | 44.67±1.53ab | 72.33±24.38a |
| 50mg/L-170nm | 96±4.18ab | 0.70±0.02bc | 40.33±6.11b | 96.00±39.67a |
| 20mg/L-330nm | 92±2.74b | 0.78±0.01ab | 43.33±6.51b | 73.00±32.05a |
| 50mg/L-330nm | 99±2.24a | 0.80±0.06a | 52.33±3.21a | 96.00±20.66a |

Note: the data in the table represent means ± SDs, and significant differences between treatment groups at the 0.05 level are indicated by different lowercase letters.
